# Very Late Recurrence in Germ Cell Tumor of the Testis: Lessons and Implications

**DOI:** 10.3390/cancers14051127

**Published:** 2022-02-23

**Authors:** Joseph A. Moore, Rebecca S. Slack, Michael J. Lehner, Matthew T. Campbell, Amishi Y. Shah, Miao Zhang, Charles C. Guo, John F. Ward, Jose A. Karam, Christopher G. Wood, Louis L. Pisters, Shi-Ming Tu

**Affiliations:** 1 Division of Cancer Medicine, The University of Texas MD Anderson Cancer Center, Houston, TX 77030, USA; joseph.moore@cancercenterofkansas.com; 2 Department of Biostatistics, The University of Texas MD Anderson Cancer Center, Houston, TX 77030, USA; rsslack@mdanderson.org; 3 Department of Internal Medicine, University of Texas Health Science Center, Houston, TX 77030, USA; michael.j.lehner@uth.tmc.edu; 4 Department of Genitourinary Medical Oncology, The University of Texas MD Anderson Cancer Center, Houston, TX 77030, USA; mcampbell3@mdanderson.org (M.T.C.); ayshah@mdanderson.org (A.Y.S.); 5 Department of Pathology, The University of Texas MD Anderson Cancer Center, Houston, TX 77030, USA; mzhang8@mdanderson.org (M.Z.); ccguo@mdanderson.org (C.C.G.); 6 Department of Urology, The University of Texas MD Anderson Cancer Center, Houston, TX 77030, USA; jfward@mdanderson.org (J.F.W.); jakaram@mdanderson.org (J.A.K.); cgwood@mdanderson.org (C.G.W.); lpisters@mdanderson.org (L.L.P.)

**Keywords:** testicular cancer, late recurrence, germ cell tumor, nonseminoma

## Abstract

**Simple Summary:**

Very late recurrence of testicular germ cell tumors, i.e., greater than 5 years after initial presentation, is a rare event occurring in about 1% of patients and is associated with poor prognosis. Our study sought to add to the available data and characterize patients with late recurrence. Patients with late recurrence > 5 years after initial presentation tend to harbor nonseminomatous germ cell tumors (with yolk sac tumor and or teratoma). Among these patients, a majority who did not undergo surgery to remove residual disease after chemotherapy developed somatic transformation and succumbed to their late recurrence. Further investigation into rates of late recurrence among all patients may be warranted given the poor survival after late recurrence.

**Abstract:**

Background. Very late recurrence (LR), i.e., >5 years after initial presentation, occurs in about 1% of patients with germ cell tumors of the testis (TGCT) and is associated with poor prognosis. Methods. We retrospectively reviewed the records of patients at the M. D. Anderson Cancer Center who developed LR > 5 years after their initial diagnosis of TGCT. Results. We identified 25 patients who developed LR between July 2007 and August 2020. The median age at the time of LR was 46 years (range, 29–61). Pathology of LR: somatic transformation to carcinoma or sarcoma—11, nonseminoma with yolk sac tumor or teratoma—11, nonseminoma without yolk sac tumor or teratoma—2, not available—1. With a median follow-up of 3.5 years, 68% of patients are alive 3 years after LR. Patients with prior post-chemotherapy consolidation surgery do not have statistically significant longer survival compared to patients who did not receive post-chemotherapy consolidation surgery, 83.3% vs. 60.8% at 3 years, respectively, *p* = 0.50. Conclusions. Patients with LR > 5 years tend to harbor nonseminoma (with yolk sac tumor and or teratoma). Among these patients, a majority who did not undergo surgery to remove residual disease after chemotherapy developed somatic transformation and succumbed to their LR.

## 1. Introduction

Germ cell tumors of the testis (TGCT) have a good prognosis with overall survival rates greater than 90%. Depending on the initial stage, about 10–30% of patients with TGCT develop recurrent disease after initial treatment and these recurrences usually occur within the first 2 years [1,2,3]. Very late recurrence (LR), i.e., >5 years after initial presentation, is a rare event occurring in about 1% of patients with TGCT and is associated with poor prognosis. Factors associated with LR include an advanced stage at diagnosis with the presence of bulky adenopathy, as well as teratoma at post-chemotherapy retroperitoneal lymph node dissection [1,2,4,5,6]. The long latency period prior to relapse presents a dilemma with regard to the recommended length of follow-up. The current guideline does not require follow-up after 5 years to detect LR, except in those presenting with metastatic nonseminomatous TGCT. There are limited data available regarding patients with LR of TGCT and many of the available series do not include patients treated with high dose chemotherapy and autologous stem cell transplantation due to poor outcomes [1,2,4,5,7,8]. In this study, we seek to add to the available data by describing our large single-center experience with LR, the clinical and pathologic features of patients with LR, as well as predictors of survival.

## 2. Patients and Methods

We retrospectively reviewed the records of 25 patients with TGCT from the Genitourinary Medical Oncology clinic at the M. D. Anderson Cancer Center (Houston, TX, USA) who presented with LR between July 2007 and August 2020. LR was defined as recurrent disease after a 5-year or greater disease-free interval after complete remission to initial therapy, in the absence of a second primary tumor in the contralateral testicle. While this patient sample may not be a consecutive series of patients, it does represent a comprehensive collection of cases during this time period. We analyzed several parameters with respect to initial presentation including age at diagnosis, histology of primary, initial stage, and initial treatment. With regard to LR presentation, we analyzed interval to recurrence, location of LR, pathology of LR, treatment of LR, and overall survival after LR.

Overall survival from the time of LR was estimated using Kaplan–Meier estimates and compared for patient subgroups with log-rank tests. Fisher’s exact test was used to compare proportions in patient subgroups. Clinical and tumor features that may be associated with survival outcome and the decision to undergo surgery for LR were tabulated and compared between those who did vs. did not receive surgery for LR. Comparisons between the two groups were made with exact Chi-square tests for patient subgroups. The differences in time to recurrence were made with the Kruskal–Wallis test. The study was approved by the Institutional Review Board. 

## 3. Results

We identified 25 patients who developed LR between July 2007 and August 2020. Table 1 outlines the baseline patient characteristics upon initial presentation and at late relapse. The median age at diagnosis of primary TCGT was 26 years (range, 15–45). At the time of diagnosis, six patients were stage I, six patients were stage II, and 13 patients were stage III. Of the 12 patients with available baseline serum tumor markers (STMs), 11 out of 12 patients (92%) had elevated STMs at initial diagnosis (Supplementary Table S1). With regard to pathology of the primary tumor, 15 patients (60%) had nonseminoma with yolk sac tumor or teratoma involvement, one patient (4%) had nonseminoma without yolk sac tumor or teratoma, and nine patients (36%) primary pathology was not available. See Supplementary Table S1 for a detailed breakdown of mixed TGCT components. Of the 19 patients with stage II–III disease, eight patients (42%) underwent post-chemotherapy consolidation surgery to remove the residual disease. 

The median age at the time of LR was 46 years (range, 29–61). The median time of LR was 16.1 years (range, 6.8–33.1 years) after diagnosis (Table 1). Of the 24 patients with available STMs at LR, 17 of 24 patients (71%) had elevated STMs at LR. Of the 11 patients with elevated STMs at baseline, 8 of the 11 patients (73%) also had elevated STMs at LR although they were not always congruent (Supplementary Table S1). With regard to the pathology of LR, nine patients (36%) had somatic transformation to carcinoma, two patients (8%) had somatic transformation to sarcoma, 11 patients (44%) had nonseminoma with yolk sac tumor or teratoma, two patients (8%) had nonseminoma without yolk sac tumor or teratoma, and one patient (4%) did not have pathology available. Interestingly, no patients in our series had necrosis or a non-viable tumor at the time of LR. Overall, five patients (20%) had LR in retroperitoneal lymph nodes alone, six patients (24%) had non-retroperitoneal nodal or pulmonary metastases, and 14 patients (56%) had non-pulmonary visceral metastases. Additionally, 15 of 25 patients (60%) had LR that involved the retroperitoneum. Of these 15 patients, two had prior retroperitoneal lymph node dissections (RPLND). Fourteen patients went on to have surgery post recurrence at a median of 0.58 years (range 0.07–6.1 years). 

**Figure 1:**
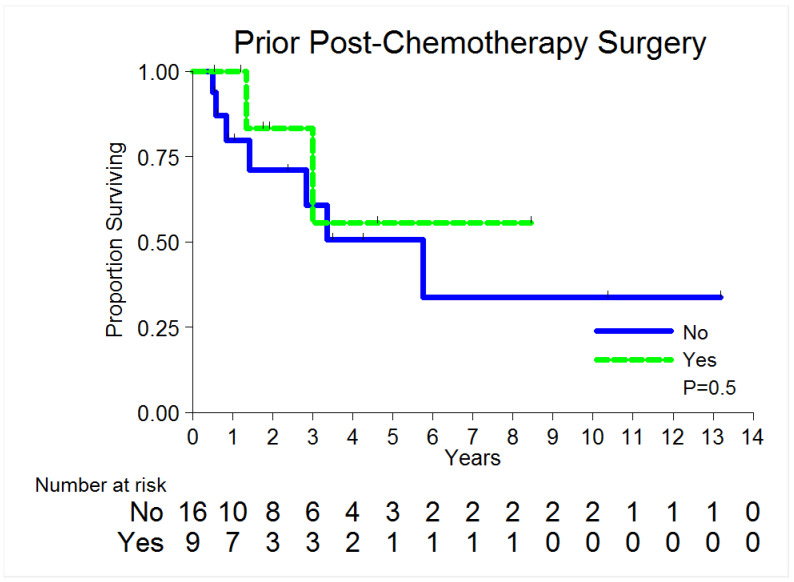
Overall survival by prior post-chemo consolidation surgery.

Table 2 displays overall survival after LR by patient and tumor characteristics. Nine patients (36%) are deceased, ten patients (40%) are alive without evidence of disease (NED), and six patients are alive with disease (24%). With a median follow-up of 3.5 years, 68% of patients are alive 3 years after LR (Table 2). Patients with prior post-chemotherapy consolidation surgery do not have statistically significant longer survival compared to patients who did not receive post-chemotherapy consolidation surgery, 83.3% vs. 60.8% at 3 years, respectively, *p* = 0.50 (Figure 1). Additionally, there was no clear association between overall survival and age of recurrence, stage of primary, pathology of primary or recurrence, and location of recurrence. 

We then analyzed the clinical and pathologic characteristics of patients broken down by surgical status after LR, i.e., comparing patients who did vs. did not receive surgery for LR (Table 3). No features were significantly associated with surgery for LR at this sample size. All five patients whose recurrence was in the RPLN alone (100%) while 43% of patients with non-pulmonary visceral metastases received surgery after LR. Patients with somatic transformation to carcinoma were most likely to get surgery (78% vs. 45–50%). Patients who did not receive chemotherapy were most likely to receive surgery (83% vs. 47%). Half of the patients who did not receive post-chemo consolidative surgery previously did receive surgery after recurrence, while 67% of patients who had prior surgery also had surgery for the recurrence. There was no difference in time to LR between those who did or did not receive surgery for LR. 

## 4. Discussion

Very late recurrence >5 years after initial diagnosis of TGCT is a rare clinical entity that affects approximately 1% of patients with TGCT. Previous studies have reported relapses more than 30 years after initial diagnosis and have suggested more than 30% of LRs may occur more than 10 years after completion of primary treatment [9]. In this study, the longest time to LR was over 33 years, which is one of the longest described late recurrences. LR can occur at any time after initial treatment of TGCT; 20 out of 25 patients (80%) in the present study relapsed after 10 years from initial diagnosis. This illustrates the importance of lifelong surveillance and follow-up in certain patients with TGCT. Guidelines from the National Comprehensive Cancer Network recommend follow-ups, including history and physical exams, serum tumor markers and imaging, with decreased frequency over time but continued beyond 5 years [10].

Unlike other malignancies where surveillance may be discontinued after 5 years, it is important to recognize a different paradigm exists with TGCT and communicate this with the patient to ensure adherence to the surveillance schedules. The current dataset emphasizes the importance of following the NCCN surveillance guidelines beyond 5 years. In our study, 71% of patients had elevated tumor markers at the time of LR. The NCCN guidelines suggest at least annual history and physical exams, as well as serum tumor markers beyond 5 years. It is also recommended to obtain computerized tomography (CT) scans of the abdomen pelvis as clinically indicated. One such clinical indication would be elevated serum tumor marker, which should be monitored closely. 

The location of recurrence seems to be important as well. The retroperitoneum is the most common site of LR with an estimated 50% of recurrences occurring in this region, followed by lung, mediastinum, neck and supraclavicular region, and pelvis [9,11]. This re-enforces the notion that control of the retroperitoneum is vitally important in the management of TGCT. Residual post-chemotherapy masses comprise necrosis in 40–50%, teratoma in 30–40%, and viable tumor in 10–20% of cases [12,13,14,15,16]. Patients with non-seminomatous TGCT who receive chemotherapy alone remain at risk for LR. Postchemotherapy retroperitoneal lymph node dissection (PC-RPLND) should be offered to patients with >1 cm residual retroperitoneal masses and should be performed in experienced centers. In our study, 15 of 25 patients (60%) had LR that involved the retroperitoneum. Of these 15 patients, two had prior RPLNDs. Several studies have suggested that relapse risk after primary or post-chemotherapy RPLND is quite low [17,18]. 

Patients with LR after chemotherapy are difficult to treat for several reasons, one of which is that the biology of LR appears to be much more aggressive and intractable than that found at the original diagnosis. The pathology of LR is more likely to be chemoresistant and comprise a yolk sac tumor and/or teratoma [19]. This is consistent with the poor outcome of patients with such pathology in the primary tumors [20,21]. The incidence of chemoresistant teratoma alone and malignant transformation of teratoma is increased in patients with LR and was found to be 16% and 44% in our patient series, respectively. Other studies have estimated the incidence of teratoma alone and malignant transformation of teratoma at about 20% and 20–25%, respectively [9,22].

Surgery is the most important part of the treatment of patients with LR and increases the chance of cure [2,4,5,8]. The histologies associated with LR, including teratoma, malignant transformation of teratoma, and chemoresistant viable TGCT, do not respond to chemotherapy and thus surgery should be the first choice of treatment for patients with the resectable disease [19,23]. Even patients with chemorefractory TGCT have a chance to be cured with “desperation surgery” to resect all visible areas of disease. It is estimated that up to 20% of patients who fit these criteria can be cured with surgical resection. Patients with isolated retroperitoneal lymph node disease, those with AFP-only elevation, and those who undergo complete resection of the residual disease have the most favorable outcome [24,25]. Referral to a center with high surgical expertise in this setting is recommended, as potentially large en-bloc resections may be required to achieve the desired outcome of complete resection [10].

The overall prognosis for patients with LR is poor with estimated long-term survival rates ranging from 25% to 60% [4,5,8,9]. Risk factors associated with improved cancer-specific survival (CSS) include complete surgical resection, single site of relapse, and asymptomatic presentation [9]. In the current study, we were unable to analyze the effect of surgery after LR for two major reasons. First, since surgery occurs after the start of survival time (i.e., date of recurrence), some of that survival time would be incorrectly attributed to surgery. Second, the decision to offer surgery to a patient is complex and depends on many factors including performance status, as well as type and location of the recurrence, all of which are also associated with survival. Therefore, we cannot separate the effect of actually performing surgery from the effect of being a surgical candidate. Analyses methods are needed to address these two issues, which require much larger sample sizes and cannot be carried out with our small group of 25 patients. 

Much like the pathogenesis of germ cell neoplasia in situ (GCNIS) and the development of testicular cancer is vaguely understood, so too is the biology of LR [26]. In LR, tumor cells may exist in a dormant state for many years prior to clinical relapse. The mechanisms that activate tumor cells from dormancy into a proliferative state remain unknown. It is conceivable that genetic mutations may occur and accumulate in the micrometastatic cells that could trigger activation from dormancy and the resultant LR, or perhaps activation from dormancy involves a change in the microenvironment of the tumor cells [27,28]. Further research is needed to investigate the biology of LR.

## 5. Conclusions

Patients with LR > 5 years after initial presentation tend to harbor nonseminoma (with yolk sac tumor and or teratoma). Among these patients, a majority who did not undergo surgery to remove residual disease after chemotherapy developed somatic transformation and succumbed to their LR. Further investigation into rates of LR among all patients may be warranted given the poor survival after LR.

## Figures and Tables

**Table 1:** Baseline patient characteristics.

Patient Characteristics	N (%)
All	25 (100%)
Age at Recurrence—median (min, max)	
N = 25	46.0 (29.0, 61.0)
Time to Recurrence—median (min years, max years)	
N = 25	16.1 (6.8, 33.1)
Stage of Primary	
I	6 (24%)
II–IIIA	12 (48%)
IIIB/IIIC	7 (28%)
Pathology of Primary	
Nonseminoma with YST or T	15 (60%)
Nonseminoma without YST or T	1 (4%)
Not available	9 (36%)
Pathology of Recurrence	
Nonseminoma with YST or T	11 (44%)
Nonseminoma without YST or T	2 (8%)
Not available	1 (4%)
Somatic transformation to carcinoma	9 (36%)
Somatic transformation to sarcoma	2 (8%)
Location of Recurrence	
RPLN alone	5 (20%)
Non-RPLN or pulm	6 (24%)
Non-pulm visceral	14 (56%)
Prior post-chemo consolidation surgery	
No	16 (64%)
Yes	9 (36%)
Post Recurrence surgery	
No	11 (44%)
Yes	14 (56%)

Legend: YST: yolk sac tumor, T: teratoma, RPLN: retroperitoneal lymph node.

**Table 2:** Overall survival after late recurrence by patient and tumor characteristics.

Patient Characteristic		Overall Survival		
		Deaths/N	3-Year (SE)	*p*-Value
All		9/25	67.6% (11.4%)	
Age at Recurrence				0.80
	25–44	4/11	69.3% (15.0%)	
	45–65	5/14	65.0% (17.6%)	
Stage of Primary				0.55
	I	2/6	83.3% (15.2%)	
	II–IIIA	4/12	33.3% (24.9%)	
	IIIB/IIIC	3/7	80.0% (17.9%)	
Pathology of Primary				0.73
	Nonseminoma with YST or T	5/15	74.2% (13.2%)	
	Nonseminoma without YST or	1/1	100.0% (0.0%)	
	Not available	3/9	54.7% (20.1%)	
Pathology of Recurrence				0.55
	Nonseminoma with YST or T	5/11	53.3% (17.6%)	
	Nonseminoma without YST or T	1/2	NA	
	Not available	0/1	100.0% (0.0%)	
	Somatic transformation to carcinoma	2/9	100.0% (0.0%)	
	Somatic transformation to sarcoma	1/2	50.0% (35.4%)	
Location of Recurrence				0.67
	RPLN alone	1/5	50.0% (35.4%)	
	Non-RPLN or pulm	2/6	62.5% (21.3%)	
	Non-pulm visceral	6/14	73.8% (13.1%)	
Prior post-chemo consolidation surgery				0.50
	No	7/16	60.8% (14.3%)	
	Yes	2/9	83.3% (15.2%)	

NA—no patients had follow-up to 3 years and the longest followed patient was alive at last contact.

**Table 3:** Clinical characteristics by surgery status after LR.

		All *		Surgery **		No Surgery Yet **		
Patient Characteristics		N	(%)	N	(%)	N	(%)	*p*-Value
All		25	(100%)	14	(56%)	11	(44%)	
Age at Recurrence								0.43
	25–44	11	(44%)	5	(45%)	6	(55%)	
	45–65	14	(56%)	9	(64%)	5	(36%)	
Elevated STMs at Recurrence *								0.08
	No	7	(29%)	6	(86%)	1	(14%)	
	Yes	17	(71%)	7	(41%)	10	(59%)	
Location of Recurrence								0.12
	Non-RPLN or pulm	6	(24%)	3	(50%)	3	(50%)	
	Non-pulm visceral	14	(56%)	6	(43%)	8	(57%)	
	RPLN alone	5	(20%)	5	(100%)	0	(0%)	
Pathology of Recurrence *								0.67
	Nonseminoma with YST or T	11	(46%)	5	(45%)	6	(55%)	
	Nonseminoma without YST or T	2	(8%)	1	(50%)	1	(50%)	
	Somatic transformation to carcinoma	9	(38%)	7	(78%)	2	(22%)	
	Somatic transformation to sarcoma	2	(8%)	1	(50%)	1	(50%)	
Chemotherapy								0.18
	No	6	(24%)	5	(83%)	1	(17%)	
	Yes	19	(76%)	9	(47%)	10	(53%)	
Stem Cell Transplant Recipient								0.29
	No	21	(84%)	13	(62%)	8	(38%)	
	Yes	4	(16%)	1	(25%)	3	(75%)	
Prior Post-Chemo Consolidative Surgery								0.68
	No	16	(64%)	8	(50%)	8	(50%)	
	Yes	9	(36%)	6	(67%)	3	(33%)	
Years from Dx to Recurrence-median (IQR)	N = 25	16.1	(13.7, 23.1)	18.7	(9.9, 23.1)	16.1	(13.7, 25.3)	0.96
Died								***
	No	16	(64%)	13	(81%)	3	(19%)	
	Yes	9	(36%)	1	(11%)	8	(89%)	

* Column for All gives percentages that sum to 100% down the column. Patients with information not available were excluded for that analysis, so column may not always sum 25 patients. ** Columns for Surgery status sum to 100% in each row in order to present the estimate of patients who received surgery in each subgroup. *** Patients can receive surgery any time after recurrence, so patients who have not had surgery at the time of analysis may still get surgery. Patients who were too sick to receive surgery did not. Because of this bias, no *p*-value is included for the association with whether the patient died. A much larger sample size is needed to model any surgical effect controlling for bias or using surgery as a time-varying covariate. Among patients without surgery, the 3-year OS estimate is 51% (SE = 16%) with the longest followed patient dying at 5.8 years. For survival post-surgery among patients with surgery, The OS estimate at 3 years is 80% (SE 18%). Only death occurred at 2.1 years post-surgery, with the longest followed patient alive at 8 years, and one patient whose last survival follow-up was the date of surgery.

## Data Availability

Data are contained within the article or supplementary material.

## Supplementary Materials

The following supporting information can be downloaded at: https://www.mdpi.com/article/10.3390/cancers14051127/s1, Table S1: Patient Characteristics at Initial Presentation and at Late Relapse.
