# Supplementary material for: Very Late Recurrence in Germ Cell Tumor of the Testis: Lessons and Implications
Source: Cancers (Basel). 2022 Feb 23;14(5):1127. doi: 10.3390/cancers14051127 (PMC8909729; doi:10.3390/cancers14051127)
Supplement: Supplementary file 1 [file cancers-14-01127-s001.zip › cancers-1534065-supplementary.pdf]

cancers MDPI

Table S1. Patient Characteristics at Initial Presentation and at Late Relapse.

Pt	Age at Dx	Stage	Primary pathology	Elevated AFP at Dx	Elevated HCG at DX	Initial Treatment (pathology if resection)	Time to relapse, years	Site of recurrence	Treatment at Relapse	Pathology of recurrence	Disease status (months from relapse)	Elevated AFP at LR	Elevated HCG at LR
1	36.4	IIIC	C + E + T + Y	NA	Yes	BEP	18.3	Bone, MN, RPLN	XRT	ST-C	D (69)	Yes	No
2	25.9	IIA	E + T + Y	NA	Yes	EP	9.3	Bone, CNS, Lungs, RPLN	VIP Thoracotomy	C	A^+ (28)	Yes	Yes
3	45.4	IS	E + S + T	Yes	Yes	CEB	16.1	Colon, RPLN	FOLFOX, TIP HDC-SCT	E + Y + ST-C	D (40)	Yes	Yes
4	22.4	IIIA	E + T + Y	NA	NA	BEP	13.3	CNS, MN, RPLN	VIP HDC-SCT	Y	D (10)	Yes	Yes
5	29.4	IA	S + T	NA	NA	XRT	8.1	RPLN	RPLND	ST-C + T	A^0 (7)	No	No
6	25.6	IIIB	C + E + T + Y	Yes	Yes	BEP, EP RPLND (T) Lung/NN resection (T)	13.6	Pleura	CAV + IE	ST-S	D (16)	No	No
7	15.9	I	NA	NA	NA	BEP RPLND (E + T) VIP, TIP	13.8	Lung, MN, pleura	Surgery TIP x4	E + T + Y	A^0 (101)	Yes	No
8	32.2	II	T	NA	NA	BEP, EP RPLND (NVT)	26.8	NN, RPLN	EP x4 RPLND	ST-C	A^+ (6)	Yes	No
9	39.7	IIIC	NA	NA	NA	BEP XRT	9.9	Bone, ILN, MN, skin	TIP RPLND HDC-SCT XRT	ST-C	A^0 (124)	NA	NA
10	19.3	IIIA	E + T + Y	Yes	Yes	BEP	21.8	Lung, RPLN	TIP	T	A^+ (9)	Yes	No
11	17.3	IIIA	E + Y (RPLN)	NA	NA	BEP	25.3	Lungs, RPLN	VeIP	E + Y	A^0 (51)	Yes	No
12	30.9	IA	E + S + Y	No	No	Surveillance	6.8	RPLN	EP RPLND	ST-S	A^0 (158)	No	No
13	24.6	II	NA	NA	NA	BEP	27.3	RPLN	RPLND TIP, ACE	Y	D (34)	Yes	No
14	34.9	IIIC	T + Y	Yes	Yes	BEP, VIP, TIP, ACE RPLND (T)	15.9	Mesentery, pelvis, psoas muscle	Pelvic and mesenteric resection	T	A^0 (14)	No	No
15	20.7	IIIA	NA	NA	NA	BEP	14.8	MN, RPLN	EP, ATP, TIP, GTA	Y	D (7)	Yes	No
16	36.9	IIIA	NA	NA	Yes	BEP	14.4	Bone, ILN, lung	TIP, ATP XRT	C + E + T	D (17)	No	Yes
17	17.3	IIIB	E	NA	Yes	VeIP RPLND (NVT)	30.2	Bone, liver, lung, RPLN	BEP, TIP FOLFOX	Y	A^+ (6)	Yes	No
18	25.6	IIIB	E	Yes	Yes	BEP, EP, POMB, ACE RPLND (NVT)	17.0	Liver, LN	ATP, TIP Temsirolimus HDC-SCT Vandetinib Lenalidomide + Bevacizumab	Y	D (36)	Yes	Yes
19	30.2	II	E + T + Y	Yes	Yes	XRT	30.5	RPLN	RPLND	Y + ST-C	A^+ (6)	No	No
20	24.4	IS	Y	NA	NA	BEP	8.8	Brain, liver, bone, lungs	TIP WBRT HDC-SCT Cabozantinib + Nivolumab GemOx	NA	A^+ (42)	NA	Yes
21	23.8	II	NA	NA	NA	Chemo (regimen NA)	22.7	Duodenum, RPLN	XRT FOLFOX RPLND	ST-C	A^0 (12)	No	No

22	23.1	IIIA	NA	NA	NA	BEP Lobectomy (path NA)	32.7	RPLN	RPLND	ST-C	A^0 (23)	No	No
23	34.9	II	T	Yes	Yes	BEP, VIP RPLND (T) NND (T) MND (T)	21.4	MN	GTA Thoracotomy + MLND	T + ST-C	A^0 (22)	Yes	Yes
24	32.6	IIIC	C + E + T + Y	Yes	Yes	BOP, POMB, ACE Lobectomy (T + Y)	14.5	Lung, bone	Pneumonectomy + MLND	Y	A^0 (55)	Yes	No
25	33.6	IA	E + Y	NA	NA	Surveillance	25.3	NN, RPLN	EP	E	D (6)	No	Yes

Pt: patient; Dx: diagnosis; C: choriocarcinoma; E: embryonal carcinoma; T: teratoma; Y: yolk sac tumor; S: seminoma; NA: not available; RPLN: retroperitoneal lymph node; BEP: bleomycin, etoposide, cisplatin; EP: etoposide, cisplatin; CEB: carboplatin, etoposide, bleomycin; XRT: radiation therapy; RPLND: retroperitoneal lymph node dissection; VIP: etoposide, ifosfamide, cisplatin; TIP: paclitaxel, ifosfamide, cisplatin; NVT: no viable disease; ACE: actinomycin, cyclophosphamide, etoposide; VeIP: vinblastine, ifosfamide, cisplatin; POMB: cisplatin, vincristine, methotrexate, bleomycin; NN: neck node dissection; MN: mediastinal node dissection; BOP: bleomycin, vincristine, cisplatin; MN: mediastinal node; CNS: central nervous system; NN: neck node; ILN: inguinal node; FOLFOX: 5-fluorouracil, oxaliplatin; HDC-SCT: high-dose chemotherapy with stem cell transplant; CAV: cyclophosphamide, doxorubicin, vincristine; IE: ifosfamide, etoposide; ATP: doxorubicin, paclitaxel, cisplatin; GTA: gemcitabine, paclitaxel, doxorubicin; WBRT: whole brain radiation therapy; GemOx: gemcitabine, oxaliplatin; ST-C: somatic transformation to carcinoma; ST-S: somatic transformation to sarcoma; A^+: alive with disease, A^0: alive with no evidence of disease, D: dead.
